# Immunonutritional Protease Inhibitors from *T. durum* and *A. sativa* Display Metabolic Similarities When Assayed on Human Macrophage-like Cells

**DOI:** 10.3390/ijms22158307

**Published:** 2021-08-02

**Authors:** Bartosz Fotschki, Aurora Garcia Tejedor, Juan Antonio Nieto Fuentes, Jose Moisés Laparra Llopis

**Affiliations:** 1 Department of Biological Function of Food, Institute of Animal Reproduction and Food Research, Polish Academy of Sciences, Tuwima 10, 10-748 Olsztyn, Poland; b.fotschki@pan.olsztyn.pl; 2 Food Science and Health Group, Valencian International University (VIU), Pintor Sorolla 21, 46002 Valencia, Spain; agarciate@universidadviu.com (A.G.T.); juanantonio.nieto@campusviu.es (J.A.N.F.); 3 Madrid Institute for Advanced Studies in Food (IMDEA Food), Ctra. Cantoblanco 8, 28049 Madrid, Spain

**Keywords:** macrophage, oat, wheat, immunity, immunonutrition

## Abstract

This study evaluated the immunonutritional effects caused by protease inhibitors from Avena sativa and *Triticum durum* to human macrophage-like cells. Macrophages were exposed (3 h) to extracts obtained from flours, and mitochondrial-associated oxygen consumption rates and inflammatory, metabolic, and proteome adaptations were quantified. Mass spectrometry ‘*m*/*z*’ signals of the extracts obtained from *T. durum* and *A. sativa* revealed molecular weights of 18–35 kDa and 16–22 kDa, respectively, for the compounds present at highest concentrations. Extracts from *T. durum* exhibited lower susceptibility to degradation by gastrointestinal enzymes than those from *A. sativa*: 9.5% vs 20.2%. Despite their different botanical origin, both extracts increased TLR4 expression. Metabolic protein levels were indicative of a decreased glycolytic to lactate flux in cell cultures upon stimulation with *A. sativa* extracts, which improved mitochondrial respiration in relation to those from *T. durum*. Principal components analysis confirmed relative similarities between immune–metabolic events triggered by immunonutritional ingredients in *T. durum* and *A. sativa*. Collectively, immunonutritional effects help to interpret the differences between both crops, worsening or improving, macrophage immune reactivity (tolerogenicity), and better control of inflammatory processes.

## 1. Introduction

Immunonutritional plant protease inhibitors (PIs) can be naturally found in different cereal [1,2,3] and pseudocereal [4,5] seeds as well as in legume seeds [6], soybeans [7], tomato [8], and potato [9]. From an immunonutritional point of view, most studies focused on PIs from wheat [1,2,3], *Avena sativa*, and most recently in *Chenopodium quinoa* and *Salvia hispanica* [4,5], where macrophages appear as the target population.

At the molecular level, it appears that phenotypic adaptations to the macrophage population derive from significant metabolic changes upon interaction with the innate immune ‘Toll-like’ receptor (TLR)-4 [1,3]. In vitro approaches to evaluate the potential susceptibility of PIs to degradation by gastrointestinal enzymes transpire in the sense of a relatively high resistance to proteolysis [1,4]. Collectively, these data support a poor digestibility for PIs as well as their absorption rate, and their major effects may be derived from their interaction with intestinal TLR4, a key player on the onset of insulin resistance that seems to occur in NAFLD [10] and HCC [4]. PIs engagement of downstream signaling varies depending on different adaptor molecules. Whereas TLR4/MyD88-dependent [1] signaling likely contributes to liver inflammation, the TLR4/TRIF-dependent signaling [3] could exert protective roles in the cellular metabolic function. Often, in the TLR4-mediated inflammatory response(s), TLR4 contributes to an exacerbated intestinal innate immune activity that appears to be dependent on a manifest mitochondrial dysfunction [11]. Otherwise, recent data on the proteome response(s) triggered by PIs from *C. quinoa* and *S. hispanica* [5] indicate that TLR4/TRIF-dependent signaling does not impair mitochondria. Overall, there is a current lack of information about the cellular response(s) to PIs from *A. sativa* and *T. durum*, worsening or improving, and their macrophage functional differentiation and function.

Recent lines of evidence highlight the contribution of innate immunity response(s) and macrophages activation to the onset and/or severity of different immune–metabolic diseases (i.e., non-alcoholic fatty liver disease—NAFLD, celiac disease) [12,13] and hepatocarcinoma [4]. Overall, these studies point to the critical role of not only intestinal macrophage, but also the peripheral monocyte population. Feeding PIs from both wheat [1,10,13] and *Avena sativa* [13] increased the hepatic M1-type (F4/80^+^) macrophage population and their infiltration rate, although to a different extent. However, PIs-induced liver inflammation had different consequences. While immunonutritional PIs from wheat enhanced liver fibrogenesis in a NAFLD model [10] and favored a higher incidence of intrahepatic nodes in hepatocarcinoma (HCC)-developing mice [13], those from *A. sativa* reduced tumor burden [4,13]. Further in vitro studies using human macrophage-like (HB8902^®^) cells showed similar cell response(s) [5] closely mimicking that observed in HCC-developing mice.

Thus, the objective of this study is to expand the knowledge concerning cellular immunometabolic response(s) in the human macrophage-like HB8902© cell line upon stimulation with immunonutritional PIs extracted from *Avena sativa* and *Triticum durum*.

## 2. Results

### 2.1. Obtention of PIs-Enriched Extracts

**Figure 1:**
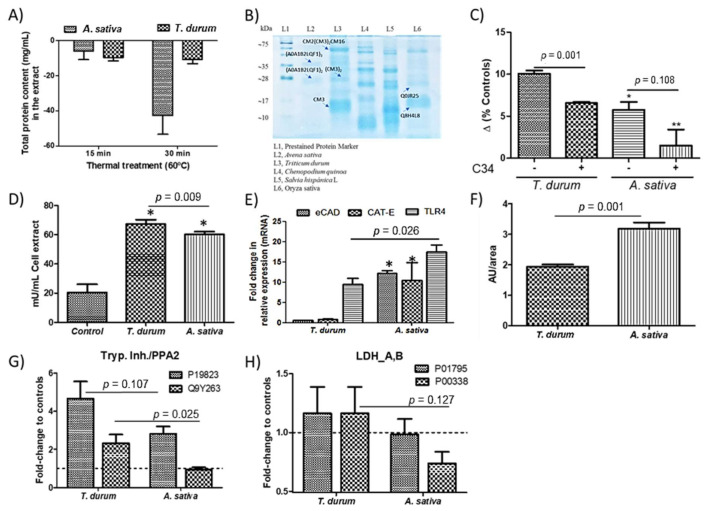
Protease inhibitors-enriched extracts. Variation of total protein content in the thermally treated extracts (**A**). Polypeptide pattern of serine-type inhibitors under SDS-PAGE separation and Coomassie staining (**B**), variations in the FACS analyses for innate immune ‘Toll-like’ receptor (TLR)-4 expression in human macrophage-like cell cultures exposed to the bioactive fraction from *T. durum* and *A. sativa* after pretreatment with the C34 TLR4 inhibitor (**C**), arginase activity assay in macrophage cell cultures upon stimulation with the extracts (**D**), variation of mRNA expression for Ca^+2^-dependent membrane adhesion cadherin-E (eCAD), endogenous protease cathepsin E (CAT-E) and TLR4. Protein changes in (**E**) expression of aryl hydrocarbon receptor (AhR) (**F**), Inter-alpha-trypsin inhibitor heavy chain (P19823) and phospholipase A2 activating protein (Q9Y263) (**G**), and L-lactate dehydrogenase isoform A (P00338) and B (P01795) (**H**). Results are expressed as mean ± SEM (*n* = 4–6). *, ** Denotes statistical differences to controls or their respective counterparts.

**Figure 2:**
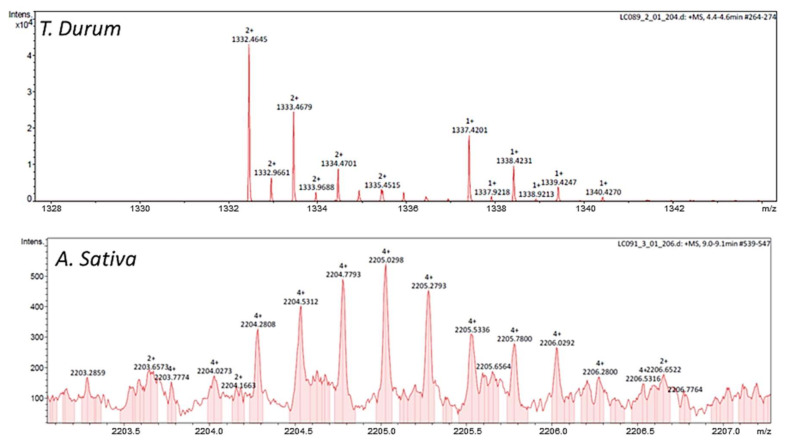
Bioactive compounds. Mass spectrometry ‘*m*/*z*’ signals of the extracts obtained from *T. durum* and *A. sativa*. Molecular masses could be calculated according to Σ[((*m*/*z*)_i_*z_i_) + ((*m*/*z*)_ii_*z_ii_) + …+((*m*/*z*)_n_*z_n_))], for ‘m’ values with variations lower to 0.5 Da.

Total protein content in the extracts from both crops was slightly higher (*p* < 0.05) from *A. sativa* (1.94 ± 0.02 mg/mL) than *T. durum* (1.66 ± 0.02 mg/mL). These contents were sequentially reduced to a different extent when treating thermally (60 °C) the extracts (Figure 1A). Electrophoresis was used to confirm the presence of bands with MW corresponding to protein complexes composed by different subunits of the PIs previously identified on these crops [1,4,5] (Figure 1B). The elimination of small proteins and peptides not associated to the albumin/globulin fraction by thermal treatment is supported by the only presence of two bands in the extract from *A. sativa* (Figure 1A,B). These bands correspond to the molecular weight of homodimeric and homotrimeric units of the serine-type PIs sequenced in *A. sativa* (A0A1B2LQF1) (www.uniprot.org, accessed on 12 March 2021). Figure 2 shows mass spectrometry ‘*m*/*z*’ signals of the bioactive subunits in the extracts obtained from *T. durum* and *A. sativa*. Overall, analyses revealed compounds with molecular weights of 18–35 kDa in *A. sativa* and 16–22 kDa in *T. durum* that were present at highest concentrations within the extracts.

After in vitro digestion, low digestibility rates were calculated: *T. durum*, 9.5%; and *A. sativa*, 20.2%. The extract from *A. sativa* significantly up-regulated TLR4 expression in cell cultures as well as that obtained from *T. durum* (Figure 1C). These cellular response(s) were interfered when cell cultures were pretreated (10 ng/mL) with C34, a TLR4 inhibitor. Notwithstanding, these effects were accompanied with increased arginase activity in cell cultures as well as (Figure 1D) increased transcripts of TLR4 (Figure 1E). However, significant (*p* < 0.05) differences appeared on the different regulation of mRNA levels of eCAD and the endopeptidase CAT-E. Besides, *A. sativa*’s extract induced an enhanced up-regulation of aryl hydrocarbon receptor (AhR) protein expression (Figure 1F) as compared with cells exposed to *T. durum*. Upon stimulation with *A. sativa*’s extract, macrophages showed up-regulated concentrations of the inter-alpha-trypsin inhibitor heavy chain (Figure 1G). Significant differences for both extracts in phospholipase A2 levels and the engagement of lactate dehydrogenase isoforms were both quantified (Figure 1H). Particularly, these changes did not occur in the isoform involved in the sub-pathway that synthesizes (*S*)-lactate from pyruvate (P01795). These changes were more prominent in the isoform modulating and cadherin binding, among others, and in response to hypoxia (P00338).

**Figure 3:**
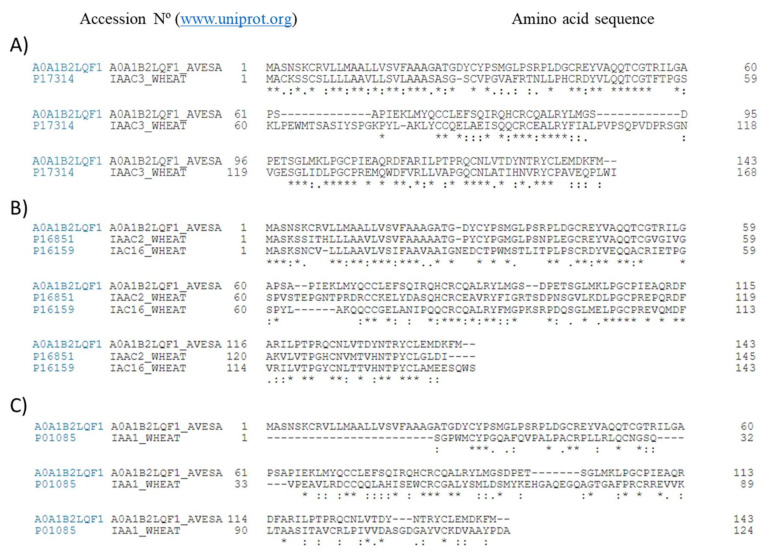
Amino acid homology. Sequence alignment between ‘serine-type’ protease inhibitors from *Avena sativa* (A0A1B2LQF1) and CM3 (P17314) from *T. aestivum* (**A**); between A0A1B2LQF1 and other protease inhibitors (CM2_P16851 and CM16_P16519) from wheat (**B**) and 0.19 (**C**) using the program CLUSTALO (www.uniprot.org, accessed on 20 April 2021). Asterisks, dot, and double dot denote identity, high homology, and low homology of amino acid residues, respectively.

These results could be partially explained by the amino acid sequence comparison of monomeric units of the serine-type PIs in both crops (Figure 3). Blast of the amino acid sequences revealed that, despite significant differences in primary amino acid sequence, both A0A1B2LQF1 and CM3 as well as other wheat PIs, as CM2 and CM16 showed highly conserved amino acid residues, suggestive of a similar secondary structure despite their botanical origin (Figure 3A,B). Cysteine residues appeared among those mostly conserved between those protease inhibitors from wheat and *A. sativa*.

### 2.2. Metabolic Changes in Macrophages

**Figure 4:**
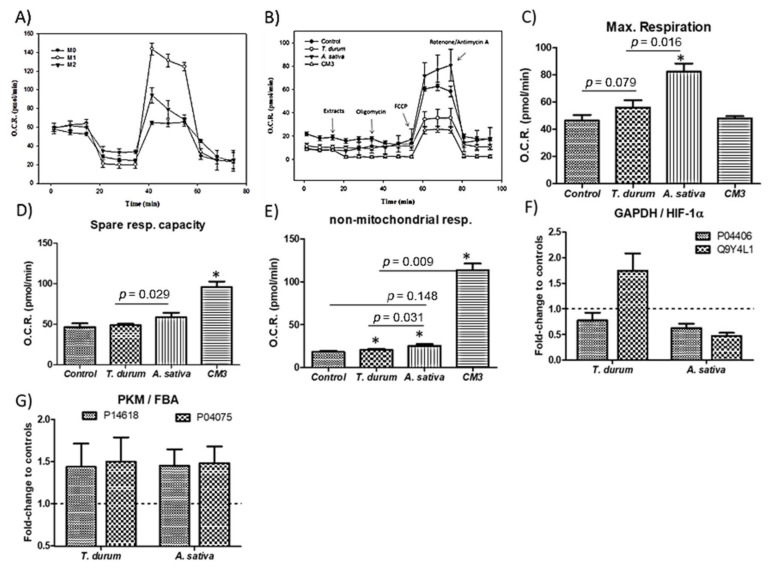
Mitochondrial stress assay. Cell Mito Stress test profile of polarized macrophages; M1 (primed with LPS + interferon-γ) and M2 (primed with IL-4 + IL-13) (**A**). Cell Mito Stress test profile of human macrophage-like cell cultures challenged to the extracts from *A. sativa* and *T. durum* (**B**). Changes in maximal respiration (**C**), spare respiratory capacity (**D**), and non-mitochondrial cell respiration (**E**). Proteome changes in expression of glyceraldehyde 3-phosphate dehydrogenase (GAPDH, P04406) and hypoxia inducible factor (HIF-1α, Q9Y4L1) (**F**), and Pyruvate kinase (PKM, P14618) and fructose-bisphosphate aldolase A (FBA, P04075) (**G**). Results are expressed as mean ± SEM (*n* = 4–6). * Denotes statistical differences to controls or their respective counterparts.

To evaluate the mitochondrial metabolic orientation of cell cultures upon stimulation with the extracts, quantified oxygen consumption rates using the Seahorse© technology were quantified (Figure 4). Macrophage metabolism has been identified as closely related to their functional differentiation state; M1 (primed with LPS + interferon-γ) and M2 (primed with IL-4 + IL-13) (Figure 4A). Profiles for the time-course of OCR variations revealed that oligomycin-induced reduction in OCR levels display an upward trend in cell cultures exposed to the extracts from both crops, and resulted similar to those obtained when challenging cell cultures to the commercial CM3 (Figure 4B). Multivariate modeling showed significant (*p* < 0.05) increases in the maximal respiration (MR) and spare respiratory capacity (SPR) of macrophages (Figure 4C–E).

These changes were accompanied with a down-regulation, similar for both extracts of the GAPDH expression (Figure 4F). In this sense, similar variations in the protein levels of pyruvate kinase (P14618) and fructose-bisphosphate aldolase A (P04075) could be quantified (Figure 4G). However, this elicited opposite changes (*p* < 0.05) on the hypoxia inducible factor (HIF)-1α production.

### 2.3. Elicit of Inflammatory Response(s)

**Figure 5:**
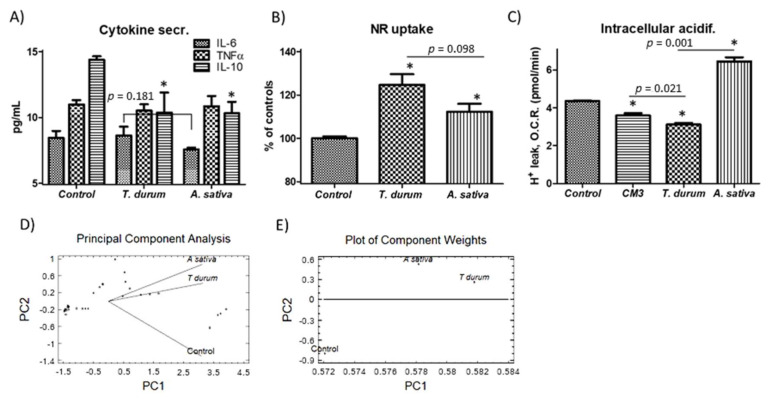
Inflammatory response(s). Cytokines secretion by macrophage cell cultures exposed to the bioactive fraction from *T. durum* and *A. sativa* (**A**), changes in endo/lysosomal activity (Neutral Red assay) (**B**) and H+ leak as an estimator of vesicular acidification (**C**). Principal component analysis (PCA) (**D**) and plot of coefficients (**E**) representing component weights (correlation coefficients between the studied factor and variables) obtained from the biochemical response(s) of macrophage cells upon stimulation with the extracts. Results are expressed as mean ± SEM (*n* = 6). * Denotes statistical differences to controls or their respective counterparts.

Extracts from both crops did not induce sharp inflammatory response(s) in human macrophage-like cells (Figure 5) upon stimulation for 3 h. The cytokines profile induced by the extracts mainly differed in the downward regulatory trend for IL-6 quantified in those cultures exposed to extracts from *A. sativa* (Figure 5A). These effects could not be attributed to proinflammatory TNFα secretion because treatment of macrophage cell cultures with both extracts did not affect TNFα secretion. Next, potential events occurring at intracellular vesicles, such as endosomes and lysosomes, were evaluated, i.e., where TLR4 localizes before retrieval to the plasmatic membrane (Figure 5B). Neutral red uptake values confirmed a similar endo/lysosomal metabolic response in cell cultures despite the origin of the extract. Otherwise, alterations in mitochondrial pH homeostasis estimated as H^+^ leak values reveal significant differences for the different extracts (Figure 5C). Under these conditions, the dynamic and fine-tuned balance in cytosolic pH that occurs in parallel to changes in the mitochondrial pH homeostasis prompts us to hypothesize differences in proton accumulation into the vesicular compartment: *A. sativa*’s extract could favor an endo/lysosomal acidification driven by V-ATPases to a higher extent than that from *T. durum*.

To synthesize the information for identifying patterns in such a way to clarify the immune–metabolic response(s) from macrophages to immunonutritional ingredients, all the data obtained were subjected to PCA (Figure 5D). PCA (2 components) plots were used for the concurrent display of biochemical and proteome changes in cells cultures subjected to the different treatments. The analyses clearly revealed separate groups of data, showing a significant biological correlation with treatment, which demonstrates the influence of the immunonutritional serine-type protease inhibitors on human macrophage-like cells.

PCA revealed a relatively similar trend for the groups of data with significant correlation upon stimulation with the extracts from both *T. durum* and *A. sativa* (Figure 5D). Here, two components could be extracted with PC#1 showing a major weight (Eigenvalue, 2.82) in relation to PC#2 (Eigenvalue, 0.11). Metabolic changes at the mitochondrial level could be determined as responsible for the major weight of PC#1. Together, these two components accounted for a cumulative percentage of 98.02% of the variability in the original data (Figure 5E).

## 3. Discussion

This study shows the presence of immunonutritional ingredients in *T. durum* and *A. sativa* that differentially modulate the immune/tolerogenicity of human macrophage-like cells. The effects derived from the challenge to both crops can contribute, worsening (*T. durum*) or improving (*A. sativa*), to the control of inflammatory processes. Previous research efforts evidenced the protease inhibitory capacity of these compounds as well as their different homo- and hetero-meric composition of the protein complexes [4] (Figure 1B, Figure 2). Even though immunonutritional compounds appear associated to minor proportions of other proteins, the immunonutritional effects could be associated to their capacity to interact with TLR4. Because of their very compact tridimensional structure, frequently due to the presence of disulfides, immunonutritional compounds are partly resistant to intestinal enzymes, keeping nondegraded immunogenic sites that can be sensed by the intestinal immune TLR4 receptor. The latter is supported by the increased TLR4 expression when cell cultures are challenged to the extracts. It was previously reported that both the extract and the dialysable (bioaccessible) fraction obtained after in vitro digestion of *T. durum* and *A. sativa* did not trigger significant changes in the production of either IL-6 or IL-10 in human macrophage-like cells (3 h) [4]. However, longer incubation times of up to 24 h favored the production of IL-8 by U937 cells [1]. Thus, as it was expected, feeding these extracts to HCC-developing mice resulted in increased proportions of hepatic F4/80^+^ cells [10,13].

Previous electrophoretic and HPLC-RP-MS analyses [13] allowed for the definition of the protein composition of the complexes found in the extracts [1,2,3,13]. A major difference between the extracts appears to imply the presence of homodimeric protein units in the extract obtained from *A. sativa*, while monomeric and heteromeric protein complexes are found in that from *T. durum*. Similarly, the presence of different subunits in elaborated extracts from wheat [2] and a CM2(CM3)2CM16 complex in seeds of *Triticum dicoccon Shrank* have been reported [14]. Previous research efforts reported homology values of 19% as indicative of identical bioactivity between wheat protease inhibitors [1]. Despite their different botanical origin, the alignment of the amino acid sequences displayed a sequence homology ≥40% for the monomeric units of protease inhibitors from *A. sativa* and *T. durum* (Figure 3). This homology could explain the stimulatory capacity of immunometabolic response(s), providing insights about their potential to interact with innate immunity contributing to influence macrophage phenotypic response(s). Accordingly, both extracts were able to interact with the TLR4 and, most probably, molecular differences between the downstream molecular events triggered by TLR4 engagement can be found.

Immunometabolic bioactivity of wheat extracts was previously determined by a HEK293 (stably transfected with the TLR4-CD14-MD2 complex) [1], cells bioassay using IL-8 secretion as readout, and the influence of TLR4 signaling inhibitor CLI-95 [3]. The immunometabolic responses detected are concordant with the production of arginase-1 in IL-4Rα^(−/−)^ and STAT6^(−/−)^ macrophages upon stimulation with LPS, as previously reported [15] (Figure 4). This response is suggestive of an intercellular communication to develop a M2-like phenotype which can limit the apparent earlier TLR4-induced inflammatory response. Furthermore, immunometabolic activity of both extracts is associated with changes in AhR, as the TLR4 ligation in LPS-primed DCs previously reported [16]. Collectively, data suggests a dampened proinflammatory responsiveness in cell cultures exposed to the extract from *A. sativa*, from which a higher rate for endopeptidase mediated degradation [17] of the protein complexes can be inferred. These effects seem to elicit a better control of inflammatory processes by *A. sativa* contributing to a more efficient resolution of chronic inflammation and tolerance. This assumption is also concordant with the role for phospholipase A2 as a key mediator of T cells recruitment, for example, amplifying inflammation [18]. The extract from *A. sativa* seemed to cause a downward trend of metabolic enzymes which are isoform-specific and are involved in the conversion of glucose into lactate. This conversion has been shown to increase in a two-stage fashion (i.e., 0–4 h and 4–12 h) after TLR (i.e., LPS, IFN-γ/LPS) M1-like activation of macrophages [19]. The molecular basis of the metabolic differences between macrophage activation by LPS/IFN-gamma or TLRs may involve a switch in the glycolytic energy metabolism, which responds to HIF-1α activation [19]. Hypoxia and glucose deprivation implies a combination of intracellular stimuli causing NLRP3 inflammasome activation [20]. The aforementioned supports that *A. sativa* extracts could contribute to the development of a tolerogenic phenotype of macrophages, perhaps via their metabolic remodeling towards regulatory metabolites of the inflammatory response [21].

Previous research efforts based on cohorts as well as short-term intervention studies have demonstrated the safe consumption of oats (uncontaminated or specially produced) by celiac disease patients, which are contradictory to studies suggesting that those may trigger exaggerated immunological response(s) in these patients [22,23]. A common aspect to all these studies is that oats do not elicit T-cell reactivity of clinical relevance. However, it is still possible that different oat cultivars may display different innate immune features relevant for the onset of inflammatory diseases affecting not only intestinal, but ‘gut-liver’ axis pathogenesis. Notably, PIs from wheat were reported to display a high innate immune bioactivity on intestinal myeloid cells [2]. Liver sections from animals subjected to a chemically induced injury displayed increased numbers of F4/80^+^ when the animals received the extracts from both *T. durum* and *A. sativa* [13]. However, this effect seemed to have a preventive influence on liver injury. These data may suggest that immune-mediated metabolic events can also contribute to establish a selective polarization of myeloid cells.

Macrophages, together with monocytes, play key roles as tissue-repairing effectors as well as gatekeepers of immune reactivity [24], although the major role is played by T cells in the development of chronic inflammation and autoimmunity [15,25,26]. Both glucose and fatty acids utilization to develop anti-inflammatory as well as inflammatory functions undergo profound metabolic reprogramming in response to intracellular metabolism alterations and hypoxia, in addition to environmental cues. Here, only extracts from *A. sativa* seemed to favor the regulatory role of macrophages on the transepithelial passage of innate and adaptive immune cells mediated by E-CAD [27]. To the best of our knowledge, only scarce data can be found regarding the microbe-mediated modulation of E-CAD transcripts as a direct contribution protecting the intestinal mucosa [28]. Thus, it could be hypothesized that the use of protease inhibitors in combined immunonutritional interventions targeting immune/tolerogenicity of intestinal macrophages may be an effective adjuvant option in the management of intestinal inflammation; for example, protection of intestinal mucosa and dampened Th1 cytokine-driven inflammation. This positive interaction can also have important consequences in reducing the intestinal cytotoxic peptides exposure because of the synergic effects between the macrophage’s cathepsin-mediated gliadin degradation [27] and gut microbiota activity [29].

Both variations in oxygen consumption rates (O.C.R.) and proteome adaptation reveal similarities between cell exposure to undigested extracts from *A. sativa* (Figure 5) and those from *Chenopodium quinoa* and *Salvia hispanica* L [5]. While the role of PIs in vivo is still under debate, a recent study demonstrated the potential of oligopeptides from *A. nuda* improving macrophage-mediated phagocytosis [30]. Otherwise, *T. durum* extracts appear to exert a negligible influence in the macrophage metabolism, impairing the intricate immune-metabolic reprogramming in macrophage activation. The metabolic adaptations derived from *A. sativa*’s extracts appear independent of the downregulation of IL-10, which predisposes to an IFNγ-induced M1 phenotype [31]. The different modulatory events caused by both extracts on the IL-6/IL-10 levels might suppose a stricter suppression of STAT3 signaling [22] by *A. sativa*’s extracts. This effect is plausible to occur, affecting calcium-dependent processes (i.e., calnexin and calcyclin) [5] that might entail the vesicular acidification and proteasomal degradation of target proteins responsible for the upregulated transcripts of TLR4 without triggering Th1 response(s). These changes are compatible with a preventive role for the lysosome of both toxic effects of undegradable protein products and abnormal immune reactivity damaging enterocytes [32].

## 4. Materials and Methods

### 4.1. Isolation of Protease Inhibitors

Commercial flours (*Avena sativa* and *Triticum durum*) were obtained from local supermarkets and processed, as described elsewhere (Laparra & Haros, 2019). Briefly, aliquots (0.5 g) were weighed into a centrifuge tube (50 mL) and extracted (×2) with 5 mL of phosphate buffered saline solution (PBS) at 37 °C with gentle agitation during 2 h. Afterwards, the suspensions were centrifuged for 15 min at 6000× *g* and supernatants subjected to thermal treatment (60 °C/30 min). After centrifugation, the clear supernatants were pooled and filtered through a 0.20 µm membrane. Total soluble protein concentrations in the extracts were determined using a micro Lowry method-based commercial kit (TP0200, Sigma, St. Louis, MO, USA). Additional reverse phase-liquid chromatography-MS/MS analyses on the extracts confirmed the molecular weight of those compounds present at highest concentrations as well as the absence of bacterial lipopolysaccharide (LPS) contamination in the extracts [13]. To ascertain potential differences between the extracts, experiments were conducted with freshly prepared solutions using the extracts normalized at a concentration of 0.1 mg protein/mL, which was chosen based on previous of our own studies [5].

### 4.2. In Vitro Digestion

Extracts from *T. durum* and *A. sativa* were subjected to a sequential simulated gastrointestinal digestion using pepsin at pH 2 (P-7000, Sigma-Aldrich, St. Louis, MO, USA) and pancreatin (P1750)/bile extract (B8631) at pH 5.5 solutions [5] to estimate their susceptibility to degradation by proteolytic enzymes. 

### 4.3. Electrophoresis

The complexity and molecular weights of isolated proteins were estimated by the SDS-PAGE analyses with 12% gels using a molecular weight marker (Cat No. B4MWP03, BlueStar Pre-stained Protein Marker, Cultek). Gels were revealed with the Coomassie reagent.

### 4.4. HPLC-Reverse Phase-Diode Array/Electrospray Ionization (HPLC-RP-DAD/ESI) Analyses of the Bioactive Fraction

The analyses of the bioactive fraction were carried out at 25 °C (0.8 mL/min) on a 1260 Agilent HPLC system equipped with a quaternary pump and a photodiode array detector set at 280 nm (Agilent Technologies, Waldbronn, Germany) [4]. The separation was performed on a Poroshell 120 (7.5 cm × 4.6 mm) C18 column (Agilent). The elution was performed using deionized water (A) and acetronitrile/trifluoroacetic acid 0.1% (*v*/*v*, B), according to the following gradients: 0–2 min, 0% B; 2.1–8 min, 5% to 30% B; 8–20 min, 30% to 50% B; 20–23 min, 50% B; 23.1–30 min, 75% B, and 30.1–35 min, 0% B. Investigation of the molecular masses was performed on Maxis II mass spectrometer with an electrospray source (Bruker Daltonics, Billerica, MA, USA). ESI ionization was performed using a Nano-bore emitters Stainless Steel ID 30 μm (Proxeon, Roskilde, Denmark) interface. Three independent samples were injected in each analysis.

### 4.5. Cell Cultures

The human macrophage-like HB-8902© cell line was obtained from the American Type Culture Collection (Rockville, MD, USA) at passage 1 and used in experiments between passages 4–8. Cell cultures were grown in Eagle’s Minimum Essential Medium (EMEM Glutamax, Gibco, Thermo Fisher Scientific, Inc., Waltham, MA, USA), supplemented with 10% fetal bovine serum (Gibco). The cells were maintained at 37 °C in 5% CO_2_ at 95% air, and the culture medium were changed every 2 days.

For experimental studies, cells were seeded at a density of 10^5^ cells/well onto 12-well plates (Costar, Cambridge, MA, USA). After 24 h of seeding, cell cultures were challenged (3 h) to freshly prepared solutions (mg protein/mL serum free EMEM) of the extracts. Untreated control cell cultures were used throughout. 

### 4.6. Macrophage Cell Enzyme Activities 

Endo/lysosomal activity (Neutral Red uptake) (TOX-4, Sigma-Aldrich, St. Louis, MO, USA) and arginase activity assay (Cat. No MAK112, Sigma-Aldrich, St. Louis, MO, USA) were quantified by using commercial kits according to the manufacturer’s instructions [5].

### 4.7. Reverse Transcriptase-qPCR Analyses

Total RNA was extracted from cell cultures with the TRI Reagent^®^ Solution (Cat No. AM9738, Invitrogen^TM^, Carlsbad, CA, USA) according to the manufacturer’s instructions. Total RNA (1 μg) was converted to double-stranded cDNA using AMV Reverse Transcriptase (Cat No. M9004 Promega, Madison, USA). PCR was performed with primers designed (www.ncbi.nlm.nih.gov, accessed on 25 March 2021) for the following *Homo sapiens* genes: TLR4 (forward 5′-TAC TGC ACA AGG TGA GGT GTT-3′, reverse 5′-TGT CTC AGC CAA CTG CCT AC-3′), cathepsin-E (forward 5′-GGC CAA CTA GAG AAG ATG GGG-3′, reverse 5′-GTT TGT AGC TCA AAG CGC CC-3′), e-cadherin (forward 5′- GAA CGC ATT GCC ACA TAC AC-3′, reverse 5′-GAA TTC GGG CTT GTT GTC AT-3′) and GAPDH (forward 5′-CCA CTC CTC CAC CTT TGA CG-3′; reverse 5′-CGC CAG ACC CTG CAC TTT TT-3′); the latter used as a housekeeping gene. The relative mRNA expression of the tested genes was calculated using the 2^-ΔCp^ method [5]. Samples of each cell culture were measured in duplicate and gene expression was expressed as fold-change.

### 4.8. Protein Expression of the Aryl Hydrocarbon Receptor (AhR)

Human macrophage-like cells were grown for 72 h to promote the production of this protein. After this time, the culture medium is removed, and the cells washed with PBS and fixed in 85% (*v*/*v*) ethanol in PBS for 10 min at 4 °C. The ethanol is then removed, and the plate allowed to air dry. Cells are permeabilized with 3-to-5-min washes in 0.05% (*v*/*v*) PBS-Triton X-100. Blocking of nonspecific sites where the antibody could bind was performed for 30 min at room temperature in a blocking solution for immunocytochemistry. At the end of this time, the antibody (PE-anti mouse/human-AhR, Cat. No. 565711, BD Biosciences, BD Pharmingen™, San Diego, CA, USA) diluted 1:50 was added in blocking solution and incubated at 4 °C in a humid chamber for 16 h. After this incubation, it was washed again in 0.05% (*v*/*v*) PBS-Triton X-100 three times for 10 min and the plates were observed and photographed in a fluorescence microscope (Zeiss, Oberkochen, Germany). Results are expressed as arbitrary units/cm^2^.

### 4.9. Cell Mitochondrial Stress Test Assay

HB-8902© cells were harvested and plated in a Seahorse 96-well plate (4 × 10^4^ cells/well) [5]. The oxygen consumption rate (OCR) deriving from mitochondrial oxidative phosphorylation was assessed using an extracellular flux analyzer (Seahorse Biosciences, Billerica, MA, USA), according to the manufacturer’s recommendation. After measuring basal respiration, ATP-linked OCR were determined by injecting oligomycin (2 μM). Carbonyl cyanide-4 (trifluoromethoxy) phenylhydrazone (FCCP), an uncoupler of the electron transport chain, was used at a concentration of 1.5 μM to determine maximal respiration rate. Rotenone (0.5 μM), an inhibitor of Complex I, and Antimycin A (0.5 μM), an inhibitor of Complex III, were used to completely inhibit mitochondrial electron transport to determine non-mitochondrial OCR. Mitochondrial basal respiration, ATP turnover, and the maximal respiration were calculated after correcting for the non-mitochondrial OCR.

### 4.10. Analysis of the Proteome Changes

An Easy-nLC II system coupled to an ion trap LTQ-Orbitrap-Velos-Pro hybrid mass spectrometer (Thermo Scientific, Hemel Hempstead, UK) was used [5]. Total protein extraction [33], in-gel digestion [34,35], peptide detection, and proteins identification were performed, according to procedures previously described [36]. Briefly, dried samples from cell cultures exposed to the extracts were dissolved in 0.1% formic acid. The peptides were concentrated (on-line) by reverse phase (RP)-HPLC using a 0.1 mm × 20 mm C18 RP precolumn (Thermo Scientific), and then separated using a 0.075 mm × 250 mm C18 RP column (Thermo Scientific) operating at 0.3 µL/min. The Orbitrap resolution was set at 30.000.

Peptides were eluted using a 120-min dual gradient and ESI ionization was performed using a Nano-bore emitters Stainless Steel ID 30 μm (Proxeon, Roskilde, Denmark) interface. Peptides were detected in survey scans from 400 to 1600 amu (1 μscan), followed by twenty data-dependent MS/MS scans (Top 20), using an isolation width of 2 u (in mass-to-charge ratio units), normalized collision energy of 35%, and dynamic exclusion applied during 30 s periods. The following constraints were used for the searches: tryptic cleavage after Arg and Lys, up to two missed cleavage sites, and tolerances of 10 ppm for precursor ions and 0.8 Da for MS/MS fragment ions and the searches were performed, allowing optional Met oxidation and Cys carbamidomethylation. Search against decoy database (integrated decoy approach) using false discovery rate (FDR) < 0.01.

Peptide identification from raw data was carried out using the SEQUEST algorithm (Proteome Discoverer 1.4, Thermo Scientific, San Jose, CA, USA). A database search was performed against uniprot-homosapiens.fasta. Only proteome data of those proteins helping to support immunometabolic events are plotted. Additional information of other identified proteins potentially relevant to this study is shown in Table S1 (Supplementary Materials). The fold-change of each protein is calculated as the ratio between protein contents estimated by the emPAI values; stress/control; emPAI = 10^PAI-1^ where PAI is defined as PAI = N_obsd_/N_obsbl_, and N_obsd_ and N_obsbl_, i.e., the number of observed peptides per protein and the number of observable peptides per protein, respectively.

### 4.11. Cytokine Production Analysis

Prior to cytokine determination, cell culture supernatants were centrifuged (6000× *g*/15 min) to get clear supernatants. Tumor necrosis factor-α (TNF-α, Cat No. 31673019), interleukin (IL)-6 (Cat No. 31670069) and interleukin (IL)-10 (Cat No. 31670109) were analyzed by ELISAs (Immunotools, Friesoythe, Germany), according to the manufacturer’s recommendations.

### 4.12. Statistical Analyses

Statistical analyses were performed using SPSS v.15 software (SPSS Inc., Chicago, IL, USA). Error bars indicate standard error mean, unless specified. To compare different conditions, the analysis of variance (ANOVA) test was used. Significance was set up at *p* < 0.05. 

The principal component analysis (PCA) was performed using Statgraphics© Plus (version 5.1) (Manugistics, Inc., Rockville, MD, USA). The different parameters were statistically analyzed to find characteristic features corresponding to the different treatments. Multiple ANOVA and Fisher’s least significant differences (LSD) were applied to establish statistically significant differences. 

## 5. Conclusions

*A. sativa* and *T. durum* provide PIs, which exhibit a relatively high resistance to proteolytic enzymes. PIs promote immune–metabolic effects on macrophage metabolic function. Such effects mediated by *A. sativa*-derived extracts are likely mediated by phenotypic adaptations on mitochondrial features skewing the glycolytic to lactate metabolic fluxes in response to hypoxia. The latter could benefit the regulatory capacity of macrophages on inflammatory processes. In this line, multivariate analysis of data revealed moderate differences between biological response(s) of macrophage cultures exposed to the different extracts from *A. sativa* and *T. durum*, which appear concordant with their capacity to interact with TLR4 and bioactivity.

## Data Availability

The data that support the findings of this study are available from the corresponding author upon reasonable request.

## Supplementary Materials

The following are available online at https://www.mdpi.com/article/10.3390/ijms22158307/s1.

## References

[1] Junker Y., Zeissig S., Kim S.-J., Barisani D., Wieser H., Leffler D.A., Zevallos V., Libermann T.A., Dillon S., Freitag T.L. (2012). Wheat amylase trypsin inhibitors drive intestinal inflammation via activation of toll-like receptor 4. J. Exp. Med..

[2] Zevallos V.F., Raker V., Tenzer S., Jimenez-Calvente C., Ashfaq-Khan M., Rüssel N., Pickert G., Schild H., Steinbrink K., Schuppan D. (2017). Nutritional Wheat Amylase-Trypsin Inhibitors Promote Intestinal Inflammation via Activation of Myeloid Cells. Gastroenterology.

[3] Kaliszewska A., Martinez V., Laparra J.M. (2016). Proinflammatory responses driven by non-gluten factors are masked when they appear associated to gliadins. Food Chem. Toxicol..

[4] Laparra J.M., Haros C.M. (2019). Plant seed protease inhibitors differentially affect innate immunity in a tumor microenvironment to control hepatocarcinoma. Food Funct..

[5] Srdić M., Ovčina I., Fotschki B., Haros C.M., Laparra J.M. (2020). C. quinoa and S. hispanica L. Seeds Provide Immunonutritional Agonists to Selectively Polarize Macrophages. Cells.

[6] Savelkoul F.H., van der Poel A.F., Tamminga S. (1992). The presence and inactivation of trypsin inhibitors, tannins, lectins and amylase inhibitors in legume seeds during germination. A review. Plant Foods Hum. Nutr..

[7] Liener I.E. (1994). Implications of antinutritional components in soybean foods. Crit. Rev. Food Sci. Nutr..

[8] Ramanarayan K., Swamy G.S. (2004). Triacontanol negatively modulates the jasmonic acid-stimulated proteinase inhibitors in tomato (*Lycopersicon esculentum*). J. Plant Physiol..

[9] Li Q., Huang L., Luo Z., Tamer T.M. (2020). Stability of trypsin inhibitor isolated from potato fruit juice against pH and heating treatment and in vitro gastrointestinal digestion. Food Chem..

[10] Ashfaq-Khan M., Aslam M., Qureshi M.A., Senkowski M.S., Yen-Weng S., Strand S., Kim Y.O., Pickert G., Schattenberg J.M., Schuppan D. (2019). Dietary wheat amylase trypsin inhibitors promote features of murine non-alcoholic fatty liver disease. Sci. Rep..

[11] Van den Bossche J., Baardman J., Otto N.A., van der Velden S., Neele A.E., van den Berg S.M., Luque-Martin R., Chen H.-J., Boshuizen M.C.S., Ahmed M. (2016). Mitochondrial Dysfunction Prevents Repolarization of Inflammatory Macrophages. Cell Rep..

[12] Alisi A., Carpino G., Oliveira F.L., Panera N., Nobili V., Gaudio E. (2017). The Role of Tissue Macrophage-Mediated Inflammation on NAFLD Pathogenesis and Its Clinical Implications. Mediators Inflamm..

[13] Laparra J., Fotschki B., Haros C. (2019). Immunonutritional consequences of different serine-type protease inhibitors in a C57BL/6 hepatocarcinoma model. Oncotarget.

[14] Capocchi A., Muccilli V., Cunsolo V., Saletti R., Foti S., Fontanini D. (2013). A heterotetrameric alpha-amylase inhibitor from emmer (Triticum dicoccon Schrank) seeds. Phytochemistry.

[15] Serena G., Huynh D., Lima R.S., Vise L.M., Freire R., Ingano L., Leonard M.M., Senger S., Fasano A. (2019). Intestinal Epithelium Modulates Macrophage Response to Gliadin in Celiac Disease. Front. Nutr..

[16] Salazar F., Awuah D., Negm O.H., Shakib F., Ghaemmaghami A.M. (2017). The role of indoleamine 2,3-dioxygenase-aryl hydrocarbon receptor pathway in the TLR4-induced tolerogenic phenotype in human DCs. Sci. Rep..

[17] Van den Bossche J., Laoui D., Naessens T., Smits H.H., Hokke C.H., Stijlemans B., Grooten J., De Baetselier P., Van Ginderachter J.A. (2015). E-cadherin expression in macrophages dampens their inflammatory responsiveness in vitro, but does not modulate M2-regulated pathologies in vivo. Sci. Rep..

[18] Bertsch T., Aufenanger J. (1997). Phospholipase A2 in inflammatory bowel disease. Gut.

[19] Rodríguez-Prados J.-C., Través P.G., Cuenca J., Rico D., Aragonés J., Martín-Sanz P., Cascante M., Boscá L. (2010). Substrate fate in activated macrophages: A comparison between innate, classic, and alternative activation. J. Immunol..

[20] Watanabe S., Usui-Kawanishi F., Karasawa T., Kimura H., Kamata R., Komada T., Inoue Y., Mise N., Kasahara T., Takahashi M. (2020). Glucose regulates hypoxia-induced NLRP3 inflammasome activation in macrophages. J. Cell. Physiol..

[21] Lampropoulou V., Sergushichev A., Bambouskova M., Nair S., Vincent E.E., Loginicheva E., Cervantes-Barragan L., Ma X., Huang S.C.-C., Griss T. (2016). Itaconate Links Inhibition of Succinate Dehydrogenase with Macrophage Metabolic Remodeling and Regulation of Inflammation. Cell Metab..

[22] Maglio M., Mazzarella G., Barone M.V., Gianfrani C., Pogna N., Gazza L., Stefanile R., Camarca A., Colicchio B., Nanayakkara M. (2011). Immunogenicity of two oat varieties, in relation to their safety for celiac patients. Scand. J. Gastroenterol..

[23] Comino I., Real A., de Lorenzo L., Cornell H., López-Casado M.Á., Barro F., Lorite P., Torres M.I., Cebolla A., Sousa C. (2011). Diversity in oat potential immunogenicity: Basis for the selection of oat varieties with no toxicity in coeliac disease. Gut.

[24] Ma W.-T., Gao F., Gu K., Chen D.-K. (2019). The Role of Monocytes and Macrophages in Autoimmune Diseases: A Comprehensive Review. Front. Immunol..

[25] Diosdado B., Wijmenga C. (2005). Molecular mechanisms of the adaptive, innate and regulatory immune responses in the intestinal mucosa of celiac disease patients. Expert Rev. Mol. Diagn..

[26] Maiuri L., Ciacci C., Ricciardelli I., Vacca L., Raia V., Auricchio S., Picard J., Osman M., Quaratino S., Londei M. (2003). Association between innate response to gliadin and activation of pathogenic T cells in coeliac disease. Lancet.

[27] Van den Bossche J., Malissen B., Mantovani A., De Baetselier P., Van Ginderachter J.A. (2012). Regulation and function of the E-cadherin/catenin complex in cells of the monocyte-macrophage lineage and DCs. Blood.

[28] Van Leeuwen M., Costes L., van Berkel L., Simons-Oosterhuis Y., du Pré F., Kozijn A., Raatgeep R., Lindenbergh-Kortleve D., van Rooijen N., Koning F. (2017). Macrophage-mediated gliadin degradation and concomitant IL-27 production drive IL-10- and IFN-γ 3-secreting Tr1-like-cell differentiation in a murine model for gluten tolerance. Mucosal Immunol..

[29] Laparra J.M., Sanz Y. (2010). Bifidobacteria inhibit the inflammatory response induced by gliadins in intestinal epithelial cells via modifications of toxic peptide generation during digestion. J. Cell. Biochem..

[30] Mao R., Wu L., Zhu N., Liu X., Liu R., Li Y. (2019). Naked Oat (Avena nuda L.) Oligopeptides:Immunomodulatory Effects on Innate and AdaptiveImmunity in Mice via Cytokine Secretion, AntibodyProduction, and Th Cells Stimulation. Nutrients.

[31] Chan L.L.Y., Cheung B.K.W., Li J.C.B., Lau A.S.Y. (2010). A role for STAT3 and cathepsin S in IL-10 down-regulation of IFN-gamma-induced MHC class II molecule on primary human blood macrophages. J. Leukoc. Biol..

[32] Peters T.J., Heath J.R., Wansbrough-Jones M.H., Foe W.F. (1975). Enzyme activities and properties of lysosomes and brush borders in jejunal biopsies from control subjects and patients with coeliac disease. Clin. Sci. Mol. Med..

[33] Laparra J.M., Alfonso-García A., Alegría A., Barberá R., Cilla A. (2015). 7keto-stigmasterol and 7keto-cholesterol induce differential proteome changes to intestinal epitelial (Caco-2) cells. Food Chem. Toxicol..

[34] Shevchenko A., Wilm M., Vorm O., Mann M. (1996). Mass spectrometric sequencing of proteins silver-stained polyacrylamide gels. Anal. Chem..

[35] Moreno M.-L., Escobar J., Izquierdo-Álvarez A., Gil A., Pérez S., Pereda J., Zapico I., Vento M., Sabater L., Marina A. (2014). Disulfide stress: A novel type of oxidative stress in acute pancreatitis. Free Radic. Biol. Med..

[36] Alonso R., Pisa D., Marina A.I., Morato E., Rábano A., Rodal I., Carrasco L. (2015). Evidence for fungal infection in cerebrospinal fluid and brain tissue from patients with amyotrophic lateral sclerosis. Int. J. Biol. Sci..

