# Supplementary material for: Immunonutritional Protease Inhibitors from T. durum and A. sativa Display Metabolic Similarities When Assayed on Human Macrophage-like Cells
Source: Int J Mol Sci. 2021 Aug 2;22(15):8307. doi: 10.3390/ijms22158307 (PMC8347451; doi:10.3390/ijms22158307)
Supplement: Supplementary file 1 [file ijms-22-08307-s001.zip › ijms-1293387-supplementary.pdf]

Supplementary Materials

Table S1. Proteins identified by RP-LC-MS/MS in human-like macrophage cells incubated with extracts from *T. durum* and *A. sativa* compared to untreated controls.

Nº (#)	Protein[a] (Accession)	Mr	pI	Mean fold difference[b]		*P* [c]
				T. durum	***A. sativa***	
1	Heat shock protein HSP 90-beta (P08238)	83.2	5.03	1.12 ± 0.51	0.88 ± 0.31	0.394
2	Pyruvate kinase (P14618)	57.9	7.84	1.28 ± 0.59	1.31 ± 0.46	0.943
3	Polyubiquitin-C (P0CG48)	77.0	7.66	1.04 ±0.48	0.85 ± 0.30	0.466
4	Transitional endoplasmic reticulum ATPase (P55072)	89.3	5.26	1.13 ± 0.52	1.20 ± 0.43	0.809
5	T-complex protein 1 subunit zeta (P40227)	58.0	6.68	1.04 ± 0.48	0.97 ± 0.34	0.793
6	6-phosphogluconate dehydrogenase (P52209-2)	51.8	7.44	0.87 ± 0.40	0.99 ± 0.35	0.619
7	Ras-related protein Rab-7a (P51149)	23.5	6.70	1.04 ± 0.48	0.97 ± 0.34	0.793
8	Aldehyde dehydrogenase (P05091-2)	51.0	6.20	0.87 ± 0.40	0.14 ± 0.05	0.004
9	D-3-phosphoglycerate dehydrogenase (O43175)	56.6	6.71	1.46 ± 1.46	1.02 ± 0.36	0.231
10	L-lactate dehydrogenase B chain (P07195)	36.6	6.05	1.04 ± 0.48	1.13 ±0.40	0.753
11	Sodium/potassium-transporting ATPase (P05023-3)	109.5	5.31	2.35 ± 1.07	1.48 ± 0.53	0.146
12	Fructose-bisphosphate aldolase A (P04075)	39.4	8.09	1.34 ± 0.61	1.33 ± 0.47	0.989
13	Annexin A5 (P08758)	35.9	5.05	1.46 ± 0.67	1.36 ± 0.48	0.793
14	Macrophage-capping protein (P40121-2)	36.8	7.20	1.04 ± 0.48	0.85 ± 030	0.467
15	Dolichyl-diphosphooligosaccharide-glycosyltransferase (P39656-3)	49.0	6.86	1.04 ± 0.48	0.68 ± 0.24	0.167
16	Nucleoside diphosphate kinase A (P15531)	17.1	6.19	1.30 ± 0.60	0.42 ± 0.15	0.013
17	T-complex protein 1 subunit delta (P50991)	57.9	7.83	0.59 ± 0.27	0.60 ± 0.21	0.944
18	Ubiquitin-like modifier-activating enzyme 1 (P22314-2)	113.7	5.60	0.62 ± 0.29	1.02 ± 0.36	0.092
19	Serine hydroxymethyltransferase (P34897-3)	53.4	8.15	1.04 ± 0.48	0.85 ± 0.30	0,466
20	Serine/threonine kinase receptor-associated protein (Q9Y3F4)	38.4	5.12	0.78 ± 0.36	0.42 ± 0.15	0,074
21	Glucose-6-phosphate isomerase (P06744)	63.1	8.32	0.69 ± 0.32	0.85 ± 0.30	0,453

22	T-complex protein 1 subunit α (P17987)	60.3	6.11	1.04 ± 0.48	1.13 ± 0.40	0,753
23	Chloride intracellular channel protein 1 (O00299)	26.9	5.17	1.04 ± 0.48	0.56 ± 0.20	0,074
24	Nascent polypeptide-associated complex subunit α (Q13765)	23.4	4.56	0.69 ± 0.32	0.85 ± 0.30	0,453
25	Rho GDP-dissociation inhibitor 1 (P52565)	23.2	5.11	2.08 ± 0.95	0.80 ± 0.30	0,025
26	Glucose-6-phosphate 1-dehydrogenase (P11413)	59.2	6.84	1.67 ± 0.76	0.68 ± 0.24	0.025
27	Glyceraldehyde-3-phosphate dehydrogenase (P04406)	36.0	8.46	0.69 ± 0.32	0.56 ± 0.20	0.467
28	Dihydrolipoyl dehydrogenase (P09622-2)	43.6	7.03	1.04 ± 0.48	0.85 ± 0.30	0.467
29	Ras-related protein Rab-10 (P61026)	22.5	8.38	1.39 ± 0.64	1.13 ± 0.40	0.467
30	V-type proton ATPase catalytic subunit A (P38606-2)	64.7	5.66	1.39 ± 0.64	1.13 ± 0.40	0.375
31	Poly(rC)-binding protein 1 (Q15365)	37.5	7.09	1.39 ± 0.36	0.85 ± 0.30	0.124
32	Peptidyl-prolyl cis-trans isomerase FKBP4 (Q02790)	51.8	5.43	0.52 ± 0.24	0.64 ± 0.23	0.453
33	Ras-related protein Rab-1A (P62820)	22.7	6.21	1.74 ± 0.79	0.85 ± 0.30	0.048
34	T-complex protein 1 subunit eta (Q99832-3)	54.8	7.68	1.39 ± 0.64	0.28 ± 0.10	0.005
35	Ras-related protein Rab-2A (P61019-2)	20.8	6.11	1.56 ± 0.71	0.85 ± 0.30	0.074
36	Isocitrate dehydrogenase [NADP] cytoplasmic (O75874)	46.6	7.01	1.56 ± 0.71	1.70 ± 0.45	0.754
37	Isocitrate dehydrogenase [NADP], mitochondrial (P48735-2)	45.2	7.75	1.04 ± 0.48	1.80 ± 0.60	0.092
38	Fermitin family homolog 3 (Q86UX7-2)	75.4	6.77	0.69 ± 0.32	0.85 ± 0.30	0.453
	Dolichyl-diphosphooligosaccharide--protein	67.7	6.06			
39	glycosyltransferase subunit 2 (P04844-2)			1.04 ± 0.48	0.85 ± 0.30	0.532
40	Receptor of activated protein C kinase 1 (P63244)	35.1	7.69	2.08 ± 0.95	2.12 ± 0.75	0.944
41	Ceruloplasmin (P00450)	122.1	5.72	1.56 ± 0.71	0.85 ± 0.30	0.074
42	Hypoxia up-regulated protein 1 (Q9Y4L1)	111.3	5.22	1.56 ± 0.71	0.42 ± 0.15	0.008
43	High mobility group protein B1 (P09429)	24.9	5.74	1.04 ± 0.48	1.27 ± 0.39	0.453
44	Cell division control protein 42 homolog (P60953)	21.2	6.55	1.56 ±0.71	1.37 ± 0.43	0.467
45	NADPH-cytochrome P450 reductase (P16435)	76.6	5.58	1.04 ± 0.48	1.22 ± 0.45	0.453
46	Glutamate dehydrogenase 1 (P00367-3)	46.5	7.08	1.56 ± 0.71	1.70 ± 0.60	0.754
47	Ras GTPase-activating-like protein IQGAP1 (P46940)	189.1	6.48	2.60 ± 1.19	0.42 ± 0.15	0.004

48	Proliferation-associated protein 2G4 (Q9UQ80-2)	38.0	7.53	1.04 ± 0.48	0.11 ± 0.04	0.002
49	Protein disulfide-isomerase A3 (P30101)	56.7	6.35	0.91 ± 0.42	0.95 ± 0.34	0.860
50	GTP-binding nuclear protein Ran (P62826)	24.4	7.49	1.39 ± 0.64	0.56 ± 0.20	0.025
51	Calnexin (P27824)	67.5	4.60	1.56 ± 0.71	0.85 ± 0.30	0.074
52	Serine/threonine-protein phosphatase 2A 55 kDa regulatory subunit B (P63151)	51.7	6.20	1.56 ± 0.71	0.85 ± 0.30	0.074
53	Apoptosis-inducing factor 1 (O95831-3)	66.3	8.94	0.69 ± 0.69	0.85 ± 0.30	0.453
54	Phosphoglycerate kinase 1 (P00558)	44.6	8.10	0.89 ± 0.89	0.97± 0.34	0.753
55	Phosphoglycerate mutase 1 (P18669)	28.8	7.18	1.19 ± 0.54	0.85 ± 0.30	0.254
56	40S ribosomal protein S3 (P23396)	26.7	9.66	0.92 ± 0.42	0.75 ± 0.27	0.466
57	Rab GDP dissociation inhibitor β (P50395)	50.6	6.47	0.26 ± 0.12	0.42 ± 0.15	0.093
58	T-complex protein 1 subunit γ (P49368)	60.5	6.49	0.13 ± 0.06	0.85 ± 0.30	0.001
59	T-complex protein 1 subunit τ (P50990-3)	51.6	5.24	0.17 ± 0.08	0.14 ± 0.05	0.467
60	Serine/threonine-protein phosphatase PP1-α (P62136)	37.5	6.33	0.83 ± 0.38	0.85 ± 0.30	0.944
61	T-complex protein 1 subunit β (P78371-2)	52.7	6.44	0.26 ± 0.12	1.06 ± 0.38	0.002
62	Serine/threonine-protein phosphatase PP1-γ (P36873)	37.0	6.54	0.78 ± 0.36	0.21 ± 0.08	0.008
63	Peroxiredoxin-1 (Q06830)	22.1	8.13	1.04 ± 0.48	0.97 ± 0.34	0.793
64	Sarcoplasmic/endoplasmic reticulum calcium ATPase 2 (P16615-2)	109.6	5.36	0.35 ± 0.16	0.85 ± 0.30	0.011
65	Plasminogen activator inhibitor 1 RNA-binding protein (Q8NC51-4)	42.4	8.44	2.08 ± 0.95	0.85 ± 0.30	0.025
66	Proliferating cell nuclear antigen (P12004)	28.8	4.69	1.56 ± 0.71	1.06 ± 0.38	0.202
67	Proteasome subunit beta type-5 (P28074)	28.5	6.92	0.26 ± 0.12	0.21 ± 0.08	0.467
68	Splicing factor, proline- and glutamine-rich (P23246)	76.1	9.44	1.74 ± 0.79	1.13 ± 0.40	0.167
69	Prostaglandin E synthase 3 (Q15185-3)	14.9	4.77	0.52 ± 0.24	0.85 ± 0.30	0.093
70	V-type proton ATPase subunit C1 (P21283)	43.9	7.46	0.52 ± 0.24	0.42 ± 0.15	0.467
71	Heat shock protein HSP 90-α (P07900)	84.6	5.02	0.87 ± 0.40	1.07 ± 0.38	0.449

72	RuvB-like 1 (Q9Y265)	42.1	6.38	1.39 ± 0.64	0.56 ± 0.20	0.025
73	Phospholipase A-2-activating protein (Q9Y263)	50.2	6.42	2.08 ± 0.95	0.85 ± 0.30	0.035
74	Ras-related protein Rab-14 (P61106)	87.1	6.37	3.12 ± 1.43	2.55 ± 0.90	0.467
75	Prolyl endopeptidase (P48147)	23.9	6.21	2.08 ± 0.95	2.32 ± 0.70	0.453
76	V-type proton ATPase subunit B (P21281)	80.6	5.86	3.12 ± 1.43	1.70 ± 0.60	0.074
77	Ras-related protein Rab-6A (P20340)	56.5	5.81	2.08 ± 0.95	0.85 ± 0.30	0.025
78	Dolichyl-diphosphooligosaccharide-protein glycosyltransferase subunit 1 (P04843)	23.6	5.54	4.17 ± 1.91	1.70 ± 0.60	0.042
79	Mitochondrial import receptor subunit TOM40 homolog (O96008-2)	68.5	6.38	2.08 ± 0.95	0.85 ± 0.30	0.025
80	Inter-α-trypsin inhibitor heavy chain H2 (P19823)	27.7	10.35	4.17 ± 1.91	2.55 ± 0.90	0.124
81	Ras GTPase-activating protein-binding protein 1 (Q13283)	106.3	6.85	2.08 ± 0.95	0.85 ± 0.30	0.025
82	Clathrin heavy chain 1 (Q00610-2)	187.8	5.69	2.29 ± 1.05	0.17 ± 0.06	0.002
83	Proteasome subunit beta type-4 (P28070)	29.2	5.97	0.43 ± 0.24	1.27 ± 0.45	0.011
84	Guanine nucleotide-binding protein G 'α' (P04899)	40.5	5.54	1.12 ± 0.51	0.88 ± 0.31	0.453

[a] Molecular mass (Mr, Da) and Swissprot accession name; [b] The fold change of each protein is calculated as ratio between emPAI values, stress/control; emPAI=10^{PAI} - 1 where PAI is defined as PAI = *N*obsd/*N*obsbl and *N*obsd and *N*obsbl are the number of observed peptides per protein and the number of observable peptides per protein, respectively; [c] *P* values calculated for statistical comparison between cell cultures exposed to extracts from *T. durum* or *A. sativa*.
